# Longitudinal Study of Retinal Structure, Vascular, and Neuronal Function in Patients With Relapsing-Remitting Multiple Sclerosis: 1-Year Follow-Up

**DOI:** 10.1167/tvst.10.6.6

**Published:** 2021-05-05

**Authors:** Qi Chen, Hong Jiang, Silvia Delgado, Jeffrey Hernandez, Diego Eduardo Alba, Giovanni Gregori, Kottil W. Rammohan, Vittorio Porciatti, Jianhua Wang

**Affiliations:** 1 School of Optometry and Ophthalmology, Wenzhou Medical University, Wenzhou, China; 2 Department of Ophthalmology, Bascom Palmer Eye Institute, University of Miami Miller School of Medicine, Miami, FL, USA; 3 Department of Neurology, University of Miami Miller School of Medicine, Miami, FL, USA

**Keywords:** multiple sclerosis, retinal tissue perfusion, volumetric vessel density, low contrast letter acuity, retinal ganglion cell, retinal nerve fiber layer

## Abstract

**Objective:**

The purpose of this study was to quantify retinal structural, vascular, and functional changes in patients with relapsing-remitting multiple sclerosis (RRMS) over 1 year.

**Methods:**

Eighty-eight eyes of 44 patients with RRMS underwent assessments of low contrast letter acuity (LCLA), retinal ganglion cell function detected by the steady-state pattern electroretinogram (PERG), axonal microstructural integrity measured as birefringence, intraretinal layer thicknesses by ultra-high-resolution optical coherence tomography (OCT), volumetric vessel density (VVD) by OCT angiography, and retinal tissue perfusion (RTP) by the Retinal Function Imager (RFI). All measurements were performed at baseline and 1-year follow-up. The impacts of disease activities and a history of optic neuritis (ON) were analyzed.

**Results:**

Compared to baseline, there were no significant differences in all variables (*P* > 0.05), except for the axonal birefringence and RTP. The birefringence's of the retinal fiber layer at the temporal and superior quadrants was significantly decreased (*P* < 0.05), whereas RTP was significantly increased (*P* < 0.05). In the subgroup with ON, significantly longer PERG latency and decreased VVD were observed at follow-up (*P* < 0.05). In patients with improved LCLA, significantly increased RTP and decreased VVD (*P* < 0.05) were also observed.

**Conclusions:**

This is the first longitudinal study that assessed the RTP and VVD, along with other retinal structural and functional parameters in MS. The recovery of retinal vascular function occurred with the improved LCLA, suggesting that these measurements may be associated with disease progression.

**Translational Relevance:**

The retinal microvascular changes could be potential biomarkers for monitoring therapeutic efficacy in MS.

## Introduction

Multiple sclerosis (MS) is a demyelinating inflammatory disorder of the central nervous system with progressive neurodegeneration in young and middle-aged adults. Relapsing-remitting MS (RRMS) is the most common type of MS (more than 80% of all MS cases). Patients with RRMS have acute relapses characterized by periods of clinical/radiological worsening, such as vision loss and motor deficits, followed by partial or complete recovery (remissions). However, persistent neurological deficits may accumulate after each relapse and progress even without relapses for some patients.^1^ The visual pathway is highly involved in MS, with up to 20% of patients with MS presenting with optic neuritis (ON).^2^ Given its highly heterogeneous clinical presentations and disease course, it is critical to identify biomarkers to monitor disease progression and therapeutic efficacy.^3^^,^^4^ Although brain magnetic resonance image (MRI) is routinely used to monitor disease progression, the clinical relevance of the changes in the MRI activity is uncertain.^5^

As the extension of the brain, the retina of the patients with MS displays inflammatory and neurodegenerative changes.^6^^–^^9^ Accumulating evidence indicates that retinal neurodegeneration mirrors brain alterations.^10^ For example, using spectral-domain optical coherence tomography (SD-OCT), the thinning of the peripapillary retinal nerve fiber layer (pRNFL) and combined ganglion cell and inner plexiform layer (GCIPL) is correlated with the degree of brain atrophy, visual and global disability, and the effect of MS disease-modifying therapies (DMTs).^6^^,^^7^^,^^11^ In addition to the retinal neuronal layer thinning, the dysfunction of retinal ganglion cell (RGC) has been detected by the steady-state pattern electroretinogram (PERG).^12^ The RNFL is known to exhibit birefringence, which is related to the microstructural integrity of pRNFL and to the normal function of the axons of ganglion cells.^13^ The loss of birefringence of the RNFL was demonstrated by polarization-sensitive OCT (PS-OCT).^14^ Besides neurodegeneration, the vascular abnormalities related to chronic inflammation are considered to play a role in MS pathophysiology.^15^ Impaired retinal tissue perfusion (RTP),^16^ and abnormally increased retinal volumetric microvascular density (VVD)^17^ were observed in patients with RRMS.

The goal of the present study was to longitudinally study the retinal microstructure, microvasculature, microcirculation, and axonal and neuronal functions in patients with RRMS to identify possible biomarkers for disease progression and therapeutic efficacy.

## Materials and Methods

This is a prospective longitudinal study of 44 patients with RRMS. The study was approved by the Institutional Review Board of the University of Miami and conducted in accordance with the Declaration of Helsinki. The patients were recruited from an ongoing observational cohort study at the MS Center of Excellence and Bascom Palmer Eye Institute of the University of Miami from April 2015 to February 2020. The diagnosis of MS was made based on the 2010, and, more recently, the 2017 revised McDonald Criteria.^18^ Prior ON was defined clinically, based on a history of acute vision loss associated with eye pain ≥ 6 months ago. Exclusion criteria included ophthalmologic or neurologic disorders (other than MS), such as macular edema, macular degeneration, glaucoma, diabetic and hypertensive retinopathy, or a refractive error greater than ± 6 diopters. All subjects were explained about the study procedures and signed informed consents.

Neuroradiology reports of brain MRIs at baseline and follow-up were used to define radiological disease activity. The presence of new or enlarging T2 hyperintense lesions and/or T1 gadolinium enhancement on MRI was defined as having active disease. No evidence of disease activity (NEDA) was defined as no clinical relapses, including ON and other ocular manifestations, no disability progression (Expanded Disability State Scale [EDSS], increased < 1.0), and no active disease on MRI.^19^

By consensus, DMTs were classified as low efficacy (interferons, Copaxone, and Aubagio), medium efficacy (Tecfidera and Gilenya), or high efficacy (Tysabri, Ocrevus, and Ofatumumab).^20^ The change of DMTs within the same efficacy level was defined as lateral change, whereas the change of DMTs to a more efficacious level is defined as DMT escalation.

Routine eye examination was conducted by a neuro-ophthalmologist (author H.J.) at baseline and follow-up visits. The eligibility was reviewed and decided during the eye examination. Patients with other systemic or ocular diseases or refractive errors of more than ± 6.00 diopters were excluded. Patients were scheduled for a baseline visit and a follow-up visit (12 ± 6 months). During the visit, all participants underwent the routine eye examination, including refraction to get the best-corrected visual acuity (BCVA), slit-lamp biomicroscopy, intraocular pressure (IOP) measurement, and fundus examination, followed by multimodal ophthalmic images. All examinations and imagings were done on the same day of the visits (baseline and follow-up).

### Visual Function Measured Using the LCLA Test

The low contrast letter acuity (LCLA; Sloan, Precision Vision, LaSalle, IL, USA) was used to measure low contrast visual acuity with the binocular testing of 2.5% and 1.25% LCLA. Testing occurred with lights off in the test room, and charts were placed on a retro-illuminated cabinet. With the best possible correction for refractive errors, the patients sat at a 2-M distance to read the letters, proceeding top to bottom and from left to right. The number of letters correctly read by the patient was recorded as the test score.^21^

### RGC Function Measured Using the PERG Test

A steady-state PERG (SS-PERG) was used. The instrumentation and settings were reported previously in detail.^12^ Briefly, the visual stimulus, a pattern of black-white horizontal square-wave gratings, was generated on a 14 × 14 cm light-emitting diode (LED) display (1.6 cycles/degree, 15.63 reversals/s, 98% contrast, 800 cd/sqm mean luminance, 25 degrees field; Jorvec Corp., Miami, FL, USA). The display LED monitor was placed at 30 cm viewing distance in a dimly lit room. The corrective lenses were worn for the viewing distance to obtain a Jaeger J1+ visual acuity. Subjects looked at the center of the stimulus, while the PERG was recorded from both eyes. The SS-PERG has a sinusoidal-like waveform whose first positive peak corresponds to the P1 wave of the standard transient PERG, and the peak-to-trough amplitude includes both P1 and N2 waves and the standard transient PERG.^22^ SS-PERG were Fourier-analyzed to extract the zero-to-peak amplitude (microvolts) and phase in degrees that was converted in latency (milliseconds).^23^

### Axonal Microstructural Integrity Measured as Birefringence Using PS-OCT

The PS-OCT device was custom built, and the instrumentation was reported previously.^14^ Briefly, this is a spectral-domain OCT with two identical spectrometers to acquire the reflectivity from the polarized light reflected from the retina and a reference mirror. The birefringence was measured as the phase retardation per unit depth (PR/UD), which is proportional to the birefringence.^14^ Because RNFL is known to exhibit birefringence, which is related to the microstructural integrity and normal function of the axons of ganglion cells.^13^ PS-OCT has been used to obtain the information about tissue integrity,^13^ which we refer to here as the axonal microstructural integrity of the retinal nerve fiber. A circular scan with a diameter of 3.5 mm centered on the optic nerve head was performed, and PR/UD data of the pRNFL was calculated in average and quadrantal measurements.^14^ One eye of each patient was tested to reduce the patient's burden of having multiple testing. The right eye was the first choice for imaging. If the right eye was not eligible, the left eye was imaged.

### Retinal Microvasculature Measured Using OCT Angiography 

The Zeiss Cirrus OCT angiography (OCTA) device with AngioFlex (Carl Zeiss Meditec, Dublin, CA, USA) was used to measure retinal microvascular density in the macula of 3 × 3 mm centered on the fovea from both eyes. Enface view vessel images, including total retinal vascular network (RVN), superficial vascular plexus (SVP), and deep vascular plexus (DVP), were exported and analyzed using the fractal analysis (TruSoft Benoit Pro 2.0; TruSoft International, Inc., St. Petersburg, FL, USA) to obtain fractal dimension (Dbox), representing the vessel density.^17^^,^^24^^,^^25^ OCTA projection errors as the image artifacts occur due to the large vessels in the superficial layer projected into the deep vascular plexus.^24^^,^^26^ Therefore, large vessels with a width > 25 µm were removed in the DVP. In addition, large vessels were also removed from the SVP. Fractal analysis was performed on the skeletonized vessel images to obtain the total retinal vascular density (RVD), superficial retinal vascular density (SVD), and deep retinal vascular density (DVD) in the 2.5-mm diameter annular zone after excluding the foveal avascular zone (diameter = 0.6 mm). VVD (unitless value) was calculated as the vessel density (i.e. Dbox) divided by the corresponding intraretinal tissue volume.^17^ The measurements included VVD in the SVP (VVDs), DVP (VVDd), and RVN (VVDr). The tissue volume of these corresponding intraretinal layers was measured in the same zone (2.5 mm in diameter) centered on the fovea using ultra-high-resolution optical coherence tomography (UHR-OCT).

### Retinal Microstructure Measured Using UHR-OCT

The UHR-OCT device was custom built and described in many previous studies.^24^^,^^27^ Briefly, this is an SD-OCT with an axial resolution of approximately 3 µm and a scan speed of 24,000 A-scans per second. The image acquisition and repeatability of segmentation were investigated and reported previously.^28^ The details of segmentation and analysis of intraretinal layers using UHR-OCT have also been reported in previous studies in healthy subjects,^24^^,^^29^ and patients with Alzheimer's disease^30^ and MS.^27^^,^^31^ In the present study, the scan was set to be 6 × 6 mm centered on the fovea (Fig.). The volumetric scan data (512 × 128 pixels) was processed using commercially available segmentation software (Orion; Voxeleron LLC, Pleasanton, CA, USA) to obtain annular thicknesses and tissue volumes (diameter 6 mm center on the fovea) of intraretinal layers from both eyes.^27^^,^^31^ Three intraretinal layers were segmented from the dataset acquired using UHR-OCT. These segmented layers included total retinal thickness (TRT), macular retinal never fiber layer (mRNFL), and GCIPL. In comparison, GCIPL and pRNFL were also scanned using the Zeiss Cirrus OCT. The Zeiss elliptical area (red dotted line) was also used to obtain the average thickness of GCIPL, which was obtained from the Zeiss GCIPL report. The pRNFL was also obtained from the Zeiss pRNFL report.

**Figure.**
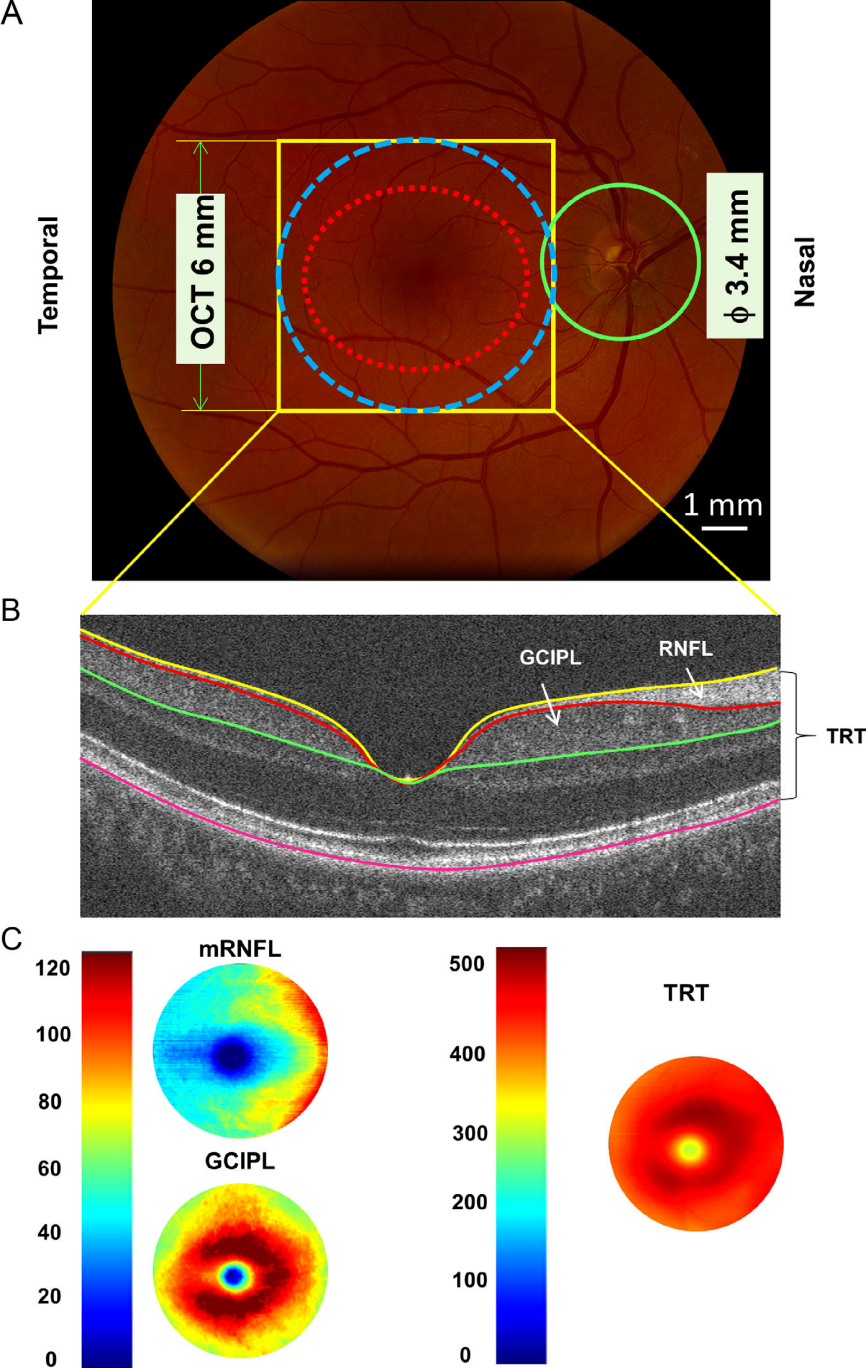
**Retinal thickness measurements using optical coherence tomography.** (**A**) UHR-OCT was used to scan the fovea with a 6 × 6 mm scan protocol (*yellow rectangular area*). The data were analyzed in the area of 6 mm disk (*blue dotted circle*) centered on the fovea to obtain tomographic thickness maps. In comparison, the Zeiss elliptical area (*red dotted line*) was also used to obtain the average thickness of ganglion cell-inner plexiform layer (GCIPL), imaged using Zeiss Cirrus OCT. In addition, the peripapillary retinal fiber layer (pRNFL, *green circle*) was also scanned using Zeiss Cirrus OCT. (**B**) Three intraretinal layers were segmented from the dataset acquired using UHR-OCT. These segmented layers included total retinal thickness (TRT), macular retinal never fiber layer (mRNFL), and ganglion cell-inner plexiform layer (GCIPL). (**C**) Intraretinal layer thickness was calculated using EDTRS partition, and annular thickness in the annulus from 1 to 6 mm in diameter was used. OCT, optical coherence tomography; UHR-OCT, ultra-high resolution optical coherence tomography, ETDRS, Early Treatment Diabetic Retinopathy Study.

### Retinal Microcirculation Measured Using Retinal Function Imager 

The retinal function imager (RFI) device (Optical Imaging Ltd, Rehovot, Israel) was used to measure retinal blood flow velocity (BFV) and retinal blood flow (RBF). The system was well-described previously.^24^^,^^29^^,^^32^^,^^33^ It applies a stroboscopic light source and a high-speed camera to capture the motion of red blood cell clusters (i.e. blood flow), and the BFV in small vessels is measured. In addition, the RBF was then calculated in all vessels entering the fovea area (diameter 2.5 mm). The RTP was calculated as the RBF divided by the corresponding tissue volume of the macula (diameter 2.5 mm) obtained by UHR-OCT, including RNFL, GCIPL, inner nuclear layer (INL), and outer plexiform layer (OPL).^16^ One eye of each patient was tested to reduce the patient's burden of having multiple testing. The right eye was the first choice for imaging. If the right eye was not eligible, the left eye was imaged.

### Statistical Analysis

Descriptive statistics and data analysis were conducted by a statistical software package (SPSS for Windows 26.0; SPSS Inc, Chicago, IL, USA). Values were presented as means ± standard deviations. Paired *t*-tests were used to determine whether there were any differences in the variables of LCLA, microcirculation, and PR/UD between baseline and after 1-year follow-up. To count inter-eye correlation and visits with the subject between subgroups, generalized estimating equation (GEE) models were used to analyze the changes of these variables between subgroups determined by LCLA, ON, disease activity, and DMT. The covariates were age, sex, and eye. In an exploratory attempt, stepwise regression was used to determine the parameters to predict the LCLA changes. In addition, binary logistic regression was also used to determine the parameters to associate the eyes with a history of ON. Any *P* < 0.05 was considered significant.

## Results

The demographic characteristics of 44 patients with MS (88 eyes) at baseline and follow-up are listed in Table 1. In 88 eyes, there were 17 eyes with remote ON. The mean follow-up duration of patients with MS was 11.8 ± 2.2 months ranged from 6 to 18 months. There were no significant differences in parameters, such as mean ocular perfusion pressure (MOPP). Color vision was full, and the confrontation visual field was normal.

**Table 1.** Demographic Characteristics of Study Subjects

	Baseline	1-y Follow-Up	*P* Values
Subjects, *n*	44	44	–
Eye, *n*	88	88	–
Age, y	40.1 ± 10.3	41.2 ± 10.3	NA
Gender, F:M	38:6	38:6	–
Follow-up duration (mo)	NA	11.8 ± 2.2	NA
SBP (mm Hg)	119.3 ± 11.8	122.4 ± 12.6	0.129
DBP (mm Hg)	79.2 ± 8.9	79.4 ± 7.9	0.872
MAP (mm Hg)	92.5 ± 9.1	93.7 ± 8.9	0.505
HR (beat per minute)	72.5 ± 11.3	72.5 ± 10.8	0.774
IOP (mm Hg)	15.5 ± 2.4	16.1 ± 2.3	0.285
MOPP (mm Hg)	51.1 ± 6.1	51.7 ± 6.0	0.774
EDSS scores	2.4 ± 1.7	2.5 ± 1.7	0.439
DD (y)	5.7 ± 6.7	6.8 ± 6.7	–

F, female; M, male; SBP, systolic blood pressure; DBP, diastolic blood pressure; MAP, mean arterial pressure = DBP + (1/3) (SBP-DBP); HR, heart rate; IOP, intraocular pressure; MOPP, mean ocular perfusion pressure = (2/3) (MAP-IOP); EDSS, Expanded Disability State Scale; DD, disease duration; -, not performed; NA, not applicable.

The details of the DMT administration over the study period are listed in Table 2. Seventeen patients had DMT escalation (newly started DMT or escalated from low efficacy to high efficacy DMT) due to clinical relapses, which happened more than 3 months before the follow-up visit. The majority of the patients met no evidence of disease activity (NEDA) status and did not change DMT or had a lateral change of DMT. Two patients on Ocrevus had increased MRI disease activity (new T2 lesions) and remained on Ocrevus.

**Table 2.** Disease-Modifying Therapy

DMT Escalation	N	DA
None to Gilenya	7	R
None to Tecfidera	1	R
Aubagio to Ofatumumab	1	R
Plegridy to Gilenya	1	R
Tecfidera to Ocrevus	1	R
Copaxone to Gilenya	2	R
Copaxone to Ocrevus	2	R
Betaseron to Gilenya	1	R
Rebif to Gilenya	1	R
**Non-DMT escalation**		
Rebif	4	NEDA
Tecfidera	7	NEDA
Gilenya	2	NEDA
Tysabri	2	NEDA
Aubagio	1	NEDA
Plegridy	2	NEDA
Copaxone	2	NEDA
None	2	NEDA
Tysabri to Ocrevus due to positive JCV	2	NEDA
Tecfidera to Gilenya due to GI	1	NEDA
Ocrevus	2	MRIa

DMT escalation, newly started or changes of disease-modifying therapy to a more potent level; Non-DMT escalation, no change or changes of disease-modifying therapy within the same potent level; N, number of patients; R, clinical relapses; NEDA, no evidence of disease activities; MRIa, magnetic resonance imaging of new T2 lesions; DA, disease activity; JCV, John Cunningham virus; GI, gastrointestinal side effects.

### Analysis of the Entire Cohort Between Baseline and Follow-Up

Compared to baseline, there were no significant differences in all variables (*P* > 0.05), except for the pRNFL birefringence and RFI measurements (Table 3). Temporal and superior PR/UD measurements were significantly decreased at follow-up (*P* < 0.05). The arteriolar and venular RBF and RTP were significantly increased at follow-up (*P* < 0.05).

**Table 3.** Comparisons of All the Variables Between Baseline and 1-Year Follow-Up in Patients With Multiple Sclerosis

Variables		Baseline (*N* = 44)	1-y Follow-Up (*N* = 44)	*P* Values
Vision function by LCLA test	**2.5% LCLA**	51.0 ± 8.7	51.6 ± 9.5	0.606
	**1.25% LCLA**	26.4 ± 10.3	26.5 ± 10.3	0.967
BCVA	**LogMAR**	0.0 ± 0.2	0.0 ± 0.2	NA
RGC function by PERG test	**Amplitude (nV)**	962.8 ± 381.9	969.1 ± 361.4	0.952
	**Latency (ms)**	50.8 ± 3.4	51.1 ± 3.7	0.818
pRNFL birefringence by PS-OCT^a^	**Average PR/UD (degree/100 µm)**	4.3 ± 2.5	3.7 ± 3.0	0.342
	**Temporal PR/UD (degree/100 µm)**	5.0 ± 3.9	3.0 ± 2.9	**0.010**
	**Superior PR/UD (degree/100 µm)**	4.1 ± 3.3	1.8 ± 2.7	**0.001**
	**Nasal PR/UD (degree/100 µm)**	7.8 ± 7.4	7.0 ± 6.1	0.588
	**Inferior PR/UD (degree/100 µm)**	5.6 ± 3.7	4.2 ± 4.5	0.088
Microvasculature by OCTA	**VVDr**	1.703 ± 0.224	1.682 ± 0.202	0.183
	**VVDs**	2.903 ± 0.511	2.867 ± 0.448	0.167
	**VVDd**	4.077 ± 0.436	4.046 ± 0.373	0.418
Microstructure by UHR-OCT	**TRT-annulus (µm)**	269.2 ± 13.6	268.6 ± 13.6	0.723
	**mRNFL-annulus (µm)**	33.6 ± 4.8	33.7 ± 4.7	0.911
	**GCIPL-annulus (µm)**	65.8 ± 7.6	65.2 ± 7.0	0.546
Microstructure by Zeiss OCT	**GCIPL (µm)**	76.3 ± 10.0	75.9 ± 9.9	**0.004**
	**pRNFL (µm)**	90.6 ± 13.1	89.9 ± 12.8	0.214
Microcirculation by RFI^a^	**Arteriolar RBF (nl/s)**	2.56 ± 0.80	2.94 ± 0.98	**0.016**
	**Venular RBF (nl/s)**	2.49 ± 0.83	2.87 ± 1.00	**0.013**
	**RTP (nl/s/mm^3^)**	2.43 ± 0.88	2.76 ± 1.00	**0.042**

LCLA, low contrast letter acuity; BCVA, best corrected high contrast visual acuity; PERG, pattern electroretinogram; mRNFL, macular retinal nerve fiber layer; pRNFL, peripapillary retinal nerve fiber layer; PS-OCT, polarization-sensitive optical coherence tomography; PR/UD, phase retardation per unit depth; OCTA, optical coherence tomography angiography; VVDr, volumetric vessel density of retinal vascular network; VVDs, volumetric vessel density of superficial vascular plexus; VVDd, volumetric vessel density of deep vascular plexus; UHR-OCT, ultra-high-resolution optical coherence tomography; TRT, total retinal thickness; RNFL, retinal nerve fiber layer; GCIPL, ganglion cell, and inner plexiform layer; RFI, retinal function imager; RBF, retinal blood flow; RTP, retinal tissue perfusion.

Bold *P* value represents < 0.05.

a Only one eye of each patient was imaged.

### Analysis of Supgroups According to LCLA

Based on the changes of the 2.5% LCLA between baseline and the follow-up, the cohort was divided into two subgroups. All retinal variables were compared between the subgroup with worsened 2.5% LCLA (*n* = 16) and the subgroup with stable or improved LCLA (*n* = 23). Except in the subgroup with significantly improved 2.5% LCLA (*P* < 0.001), there were no significant differences of all the variables between the two subgroups (*P* > 0.05; Table 4).

**Table 4.** Comparisons of the Differences of all the Variables Between Baseline and 1-Y Follow-Up According to the Changes of 2.5% and 1.25% LCLA in Patients With Multiple Sclerosis

		Δ 2.5% LCLA			Δ 1.25% LCLA		
Δ Variables		<0 (*n* = 16)	≥ 0 (*n* = 23)	*P* Values	<0 (*n* = 17)	≥ 0 (*n* = 22)	*P* Values
Clinical index	**EDSS scores**	0.1 ± 0.2	0.0 ± 0.4	0.257	0.1 ± 0.5	0.0 ± 0.2	0.639
	**MOPP (mm Hg)**	−0.2 ± 5.9	−0.4 ± 6.1	0.907	0.2 ± 7.5	−0.7 ± 4.4	0.673
**Vision function by LCLA test**	**2.5% LCLA**	−5.3 ± 4.6*	4.7 ± 5.7*	**<0.001**	−1.8 ± 7.0	2.5 ± 6.9	0.054
	**1.25% LCLA**	−1.8 ± 7.6	1.1 ± 5.3	0.181	−5.5 ± 4.0*	4.1 ± 4.5*	**<0.001**
**RGC function by PERG test**	**Amplitude (nV)**	−29.8 ± 293.8	12.3 ± 439.3	0.689	−114.2 ± 432.8	79.4 ± 324.9	0.097
	**Latency (ms)**	0.2 ± 3.0	−0.6 ± 3.1	0.372	−0.8 ± 3.6	0.1 ± 2.6	0.428
**pRNFL birefringence by PS-OCT** **^b^**	**Average PR/UD (degree/100 µm)**	−0.5 ± 4.3	−1.2 ± 4.0	0.648	−0.2 ± 4.4	−1.5 ± 3.8	0.353
	**Temporal PR/UD (degree/100 µm)**	−1.6 ± 5.5	−**2.4 ± 4.5****^a^**	0.651	−**3.4 ± 4.0****^a^**	−0.9 ± 5.4	0.109
	**Superior PR/UD (degree/100 µm)**	−1.6 ± 5.8	−**2.1 ± 3.1****^a^**	0.756	−**2.7 ± 3.3****^a^**	−1.3 ± 5.0	0.306
	**Nasal PR/UD (degree/100 µm)**	−3.8 ± 10.0	−1.0 ± 8.7	0.384	−3.8 ± 9.6	−0.8 ± 8.9	0.326
	**Inferior PR/UD (degree/100 µm)**	−**2.9 ± 5.1****^a^**	−0.7 ± 5.6	0.220	−2.9 ± 5.9	−0.5 ± 4.9	0.181
**Microvasculature by OCTA**	**VVDr**	−0.030 ± 0.142	−0.030 ± 0.138	0.982	−0.003 ± 0.134	−**0.053 ± 0.140****^a^**	0.165
	**VVDs**	−0.104 ± 0.271	−0.028 ± 0.273	0.307	0.015 ± 0.230	−**0.131 ± 0.293****^a^**	**0.045**
	**VVDd**	0.004 ± 0.388	−0.108 ± 0.407	0.258	−0.037 ± 0.414	−0.079 ± 0.393	0.694
**Microstructure by UHR-OCT**	**TRT-annulus (µm)**	−0.5 ± 6.4	0.9 ± 5.2	0.335	0.1 ± 5.6	0.5 ± 5.8	0.762
	**mRNFL-annulus (µm)**	0.2 ± 2.2	0.4 ± 3.0	0.725	0.1 ± 2.4	0.5 ± 2.9	0.504
	**GCIPL-annulus (µm)**	0.7 ± 3.1	−0.6 ± 3.5	0.076	0.3 ± 3.3	−0.4 ± 3.5	0.413
**Microstructure**	**GCIPL (µm)**	−0.3 ± 1.4	−**0.6 ± 2.0****^a^**	0.509	−0.3 ± 1.3	−**0.7 ± 2.0****^a^**	0.271
**by Zeiss OCT**	**pRNFL (µm)**	−1.1 ± 3.8	−0.2 ± 4.3	0.345	−0.6 ± 3.1	−0.6 ± 4.8	0.942
**Microcirculation by RFI** **^b^**	**Arteriolar RBF (nl/s)**	0.37 ± 0.96	**0.55 ± 1.13^a^**	0.638	0.27 ± 1.17	**0.63 ± 0.99^a^**	0.343
	**Venular RBF (nl/s)**	0.28 ± 0.93	**0.56 ± 1.18** **^a^**	0.441	0.19 ± 1.23	**0.65 ± 0.98** **^a^**	0.241
	**RTP (nl/s/mm^3^)**	0.22 ± 1.14	**0.46 ± 1.03** **^a^**	0.549	0.17 ± 1.29	**0.52 ± 0.87** **^a^**	0.380

EDSS, Expanded Disability State Scale; LCLA, low contrast letter acuity; PERG, pattern electroretinogram; mRNFL, macular retinal nerve fiber layer; pRNFL, peripapillary retinal nerve fiber layer; PR/UD, phase retardation per unit depth; OCTA, optical coherence tomography angiography; VVDr, volumetric vessel density of retinal vascular network; VVDs, volumetric vessel density of superficial vascular plexus; VVDd, volumetric vessel density of deep vascular plexus; UHR-OCT, ultra-high-resolution optical coherence tomography; TRT, total retinal thickness; RNFL, retinal nerve fiber layer; GCIPL, ganglion cell, and inner plexiform layer; RFI, retinal function imager; RBF, retinal blood flow; RTP, retinal tissue perfusion. Δ, differences between baseline and 1-year follow-up.

a With bold values, *P* < 0.05 baseline versus 1-year follow-up. Bold *P* value represents < 0.05.

b Only one eye of each patient was imaged.

In the subgroup with worsened 2.5% LCLA, the inferior PR/UD was significantly decreased at the follow-up visit (*P* < 0.05). In the subgroup with stable or improved 2.5% LCLA, a significant decrease of temporal and superior PR/UD, and significant increases of arteriolar and venular RBF and RTP were observed at the follow-up visit (*P* < 0.05).

The patients were also divided into two subgroups according to the change of 1.25% LCLA, resulting in 17 patients with worsened 1.25% LCLA and 22 with stable or improved 1.25% LCLA. There was a significant decline of VVDs in the stable or improved 1.25% LCLA subgroup compared with the worsened 1.25% LCLA subgroup (*P* = 0.045 and *P* = 0.035, respectively).

In the subgroup with worsened 1.25% LCLA, a significant decline of temporal and superior PR/UD were found at the follow-up visit, and also significant decreases of VVDr and VVDs, as well as significantly increased arteriolar and venular RBF and RTP in the subgroup with stable or improved 1.25% LCLA were observed at the follow-up visit (*P* < 0.05). Stepwise regression yielded no parameters entered in the equation to predict the LCLA.

### Analysis of Subgroups According to the History of Optic Neuritis 

Based on the remote history of optic neuritis (ON; 6 months to 11 years), 88 eyes were divided into ON (17 eyes) and non-ON (71 eyes) subgroups, respectively. There were significant changes of 2.5% LCLA (*P* = 0.038) and PERG latency (*P* < 0.001) between these two subgroups (Tables 5, 6).

**Table 5.** Comparisons of all the Variables Between Baseline and 1 Year Follow-Up in Multiple Sclerosis Patients With and Without ON Eyes

		Baseline			1-y Follow-Up		
Variables		ON (17 Eyes)	NON (71 Eyes)	*P* Values	ON (17 Eyes)	NON (71 Eyes)	*P* Values
**Vision function by LCLA test**	**2.5% LCLA**	48.9 ± 12.8	51.5 ± 7.3	0.557	47.6 ± 13.6	52.6 ± 8.0	0.224
	**1.25% LCLA**	21.5 ± 13.0	27.7 ± 9.2	0.141	22.4 ± 14.1	27.6 ± 9.0	0.200
**RGC function by PERG test**	**Amplitude (nV)**	719.0 ± 248.2	1027.8 ± 386.4	**<0.001** **^a^**	699.0 ± 271.4	1033.6 ± 351.6	**<0.001** **^a^**
	**Latency (ms)**	48.4 ± 4.5	51.4 ± 2.8	**<0.001** **^a^**	51.0 ± 5.7	51.1 ± 3.0	0.956
**pRNFL birefringence by PS-OCT** **^b^**	**Average PR/UD (degree/100 µm)**	3.9 ± 2.7	4.4 ± 2.5	0.530	3.5 ± 4.2	3.7 ± 2.5	0.880
	**Temporal PR/UD (degree/100 µm)**	3.3 ± 3.4	5.6 ± 3.9	0.051	2.3 ± 2.7	3.3 ± 3.0	0.299
	**Superior PR/UD (degree/100 µm)**	2.7 ± 4.4	4.6 ± 2.7	0.151	1.0 ± 3.6	2.2 ± 2.3	0.309
	**Nasal PR/UD (degree/100 µm)**	7.3 ± 7.8	8.0 ± 7.3	0.798	7.6 ± 5.8	6.8 ± 6.3	0.728
	**Inferior PR/UD (degree/100 µm)**	5.5 ± 4.2	5.6 ± 3.6	0.932	2.7 ± 5.8	4.8 ± 3.8	0.263
**Microvasculature by OCTA**	**VVDr**	1.915 ± 0.293	1.653 ± 0.173	**0.006** **^a^**	1.830 ± 0.276	1.649 ± 0.166	0.082
	**VVDs**	3.429 ± 0.658	2.781 ± 0.384	**0.002** **^a^**	3.210 ± 0.603	2.787 ± 0.366	**0.025** **^a^**
	**VVDd**	4.287 ± 0.609	4.028 ± 0.374	0.163	4.180 ± 0.484	4.016 ± 0.341	0.381
**Microstructure by UHR-OCT**	**TRT-annulus (µm)**	255.7 ± 15.8	272.3 ± 10.9	**<0.001** **^a^**	257.2 ± 15.9	271.4 ± 11.4	**<0.001** **^a^**
	**mRNFL-annulus (µm)**	28.9 ± 5.2	34.8 ± 4.0	**<0.001** **^a^**	29.2 ± 5.5	34.8 ± 3.7	**<0.001** **^a^**
	**GCIPL-annulus (µm)**	57.3 ± 7.7	67.8 ± 6.0	**<0.001** **^a^**	58.4 ± 7.2	66.9 ± 5.9	**<0.001** **^a^**
**Microstructure by Zeiss OCT**	**GCIPL (µm)**	65.9 ± 9.7	78.9 ± 8.3	**<0.001** **^a^**	65.4 ± 10.1	78.4 ± 8.1	**<0.001** **^a^**
	**pRNFL (µm)**	79.3 ± 11.5	93.5 ± 11.9	**<0.001** **^a^**	80.7 ± 12.0	92.1 ± 12.0	**<0.001** **^a^**
**Microcirculation by RFI** **^b^**	**Arteriolar RBF (nl/s)**	2.33 ± 0.75	2.62 ± 0.81	0.280	2.79 ± 0.78	2.98 ± 1.03	0.549
	**Venular RBF (nl/s)**	2.22 ± 0.73	2.55 ± 0.85	0.222	2.65 ± 0.58	2.93 ± 1.08	0.307
	**RTP (nl/s/mm^3^)**	2.57 ± 1.18	2.39 ± 0.81	0.657	2.92 ± 0.92	2.72 ± 1.03	0.588

ON, optic neuritis; EDSS, Expanded Disability State Scale; LCLA, low contrast letter acuity; PERG, pattern electroretinogram; pRNFL, peripapillary retinal nerve fiber layer; PS-OCT, polarization-sensitive optical coherence tomography; PR/UD, phase retardation per unit depth; OCTA, optical coherence tomography angiography; VVDr, volumetric vessel density of retinal vascular network; VVDs, volumetric vessel density of superficial vascular plexus; VVDd, volumetric vessel density of deep vascular plexus; UHR-OCT, ultra-high-resolution optical coherence tomography; TRT, total retinal thickness; RNFL, retinal nerve fiber layer; GCIPL, ganglion cell inner plexiform layer; RFI, retinal function imager; RBF, retinal blood flow; RTP, retinal tissue perfusion.

Δ, differences between baseline and 1-year follow-up.

a With bold values, *P* < 0.05 baseline versus 1-year follow-up. Bold *P* value represents < 0.05.

b Only one eye of each patient was imaged.

**Table 6.** Comparisons of the Differences of All the Variables Between Baseline and 1 Year Follow-Up in Patients With Multiple Sclerosis With and Without ON Eyes

		ON Eyes Versus Non-On Eyes		
Δ Variables		ON (17 Eyes)	Non-On (71 Eyes)	*P* Values
**Clinical index**	**EDSS scores**	0.0 ± 0.1	0.1 ± 0.4	0.208
	**MOPP (mm Hg)**	−1.6 ± 6.3	0.1 ± 5.9	0.451
**Vision function by LCLA test**	**2.5% LCLA**	−2.1 ± 6.2	1.3 ± 7.4	**0.038**
	**1.25% LCLA**	0.4 ± 7.6	−0.2 ± 6.1	0.741
**RGC function by PERG test**	**Amplitude (nV)**	−17.7 ± 267.3	−2.6 ± 406.3	0.503
	**Latency (ms)**	**2.1 ± 3.1** **^a^**	−0.9 ± 2.8	**<0.001**
**pRNFL birefringence by PS-OCT** **^b^**	**Average PR/UD (degree/100 µm)**	−0.4 ± 5.5	−0.8 ± 3.5	0.799
	**Temporal PR/UD (degree/100 µm)**	−1.0 ± 5.1	−**2.1 ± 4.7****^a^**	0.510
	**Superior PR/UD (degree/100 µm)**	−1.7 ± 6.0	−**2.0 ± 3.2****^a^**	0.851
	**Nasal PR/UD (degree/100 µm)**	0.2 ± 10.4	−1.6 ± 9.2	0.608
	**Inferior PR/UD (degree/100 µm)**	−2.7 ± 6.9	−1.0 ± 4.5	0.425
**Microvasculature by OCTA**	**VVDr**	−**0.078 ± 0.221****^a^**	−0.014 ± 0.110	0.292
	**VVDs**	−0.185 ± 0.356	−0.025 ± 0.235	0.168
	**VVDd**	−0.138 ± 0.642	−0.027 ± 0.329	0.481
**Microstructure by UHR-OCT**	**TRT-annulus (µm)**	1.2 ± 6.4	0.0 ± 5.4	0.484
	**mRNFL-annulus (µm)**	0.3 ± 2.3	0.3 ± 2.7	0.885
	**GCIPL-annulus (µm)**	0.7 ± 1.8	−0.2 ± 3.6	0.145
**Microstructure by Zeiss OCT**	**GCIPL (µm)**	−0.9 ± 2.8	−**0.4 ± 1.2****^a^**	0.482
	**pRNFL (µm)**	1.0 ± 5.5	−**1.0 ± 3.6****^a^**	0.125
**Microcirculation by RFI** **^b^**	**Arteriolar RBF (nl/s)**	0.43 ± 0.81	0.44 ± 1.13	0.980
	**Venular RBF (nl/s)**	0.40 ± 0.81	0.42 ± 1.16	0.945
	**RTP (nl/s/mm^3^)**	0.34 ± 1.19	0.33 ± 1.02	0.979

ON, optic neuritis; EDSS, Expanded Disability State Scale; LCLA, low contrast letter acuity; PERG, pattern electroretinogram; mRNFL, macular retinal nerve fiber layer; pRNFL, peripapillary retinal nerve fiber layer; PS-OCT, polarization-sensitive optical coherence tomography; PR/UD, phase retardation per unit depth; OCTA, optical coherence tomography angiography; VVDr, volumetric vessel density of retinal vascular network; VVDs, volumetric vessel density of superficial vascular plexus; VVDd, volumetric vessel density of deep vascular plexus; UHR-OCT, ultra-high-resolution optical coherence tomography; TRT, total retinal thickness; RNFL, retinal nerve fiber layer; GCIPL, ganglion cell inner plexiform layer; RFI, retinal function imager; RBF, retinal blood flow; RTP, retinal tissue perfusion.

Δ, differences between baseline and 1-year follow-up.

a With bold values, *P* < 0.05 baseline versus 1-year follow-up. Bold *P* value represents < 0.05.

b Only one eye of each patient was imaged.

In the ON subgroup, significantly longer PERG latency and decreased VVDr were observed at the follow-up visit (*P* < 0.05). In the non-ON subgroup, the changes between the baseline and 1-year follow-up of temporal and superior PR/UD were also significant (*P* < 0.05). Binary logistic regression yielded that no parameters were entered in the equation to predict the eyes with a history of ON.

### Analysis of Subgroups According to Disease Activity

According to disease activity during the study period, the cohort was divided into two subgroups (NEDA vs. non-NEDA). There were 25 patients in NEDA status and 19 with either clinical relapses and/or radiological disease activity. There were no significant differences in the changes in all variables between these two subgroups (*P* > 0.05; Table 7).

**Table 7.** Comparisons of the Differences of All the Variables Between Baseline and 1 Year Follow-Up in Patients With Multiple Sclerosis With Disease Activity

		Disease Activity		
Δ Variables		NEDA (*n* = 25)	Activity (*n* = 19)	*P* Values
**Clinical index**	**EDSS scores**	0.0 ± 0.4	0.1 ± 0.3	0.340
	**MOPP (mm Hg)**	−1.6 ± 6.6	1.3 ± 4.6	0.139
**Vision function by LCLA test**	**2.5% LCLA**	−0.2 ± 8.7	1.6 ± 4.6	0.409
	**1.25% LCLA**	−0.8 ± 6.5	0.9 ± 6.2	0.386
**RGC function by PERG test**	**Amplitude (nV)**	52.3 ± 373.8	−80.7 ± 379.7	0.224
	**Latency (ms)**	−0.4 ± 2.6	−0.1 ± 3.6	0.880
**pRNFL birefringence by PS-OCT** **^b^**	**Average PR/UD (degree/100 µm)**	−0.2 ± 4.1	−1.1 ± 4.2	0.485
	**Temporal PR/UD (degree/100 µm)**	−1.7 ± 4.7	−2.0 ± 4.9	0.833
	**Superior PR/UD (degree/100 µm)**	−1.8 ± 4.9	−**2.1 ± 3.0****^a^**	0.819
	**Nasal PR/UD (degree/100 µm)**	−0.5 ± 9.9	−1.7 ± 9.0	0.665
	**Inferior PR/UD (degree/100 µm)**	−1.5 ± 5.3	−1.5 ± 5.3	0.990
**Microvasculature by OCTA**	**VVDr**	−0.032 ± 0.154	−0.019 ± 0.116	0.761
	**VVDs**	−0.073 ± 0.298	−0.036 ± 0.229	0.636
	**VVDd**	−0.053 ± 0.401	−0.042 ± 0.407	0.937
**Microstructure by UHR-OCT**	**TRT-annulus (µm)**	0.3 ± 5.1	0.2 ± 6.2	0.982
	**mRNFL-annulus (µm)**	0.3 ± 2.0	0.3 ± 3.2	0.920
	**GCIPL-annulus (µm)**	0.2 ± 3.4	−0.2 ± 3.3	0.585
**Microstructure by Zeiss OCT**	**GCIPL (µm)**	−0.3 ± 1.1	−0.7 ± 2.3	0.247
	**pRNFL (µm)**	−0.2 ± 3.4	−1.2 ± 4.9	0.326
**Microcirculation by RFI** **^b^**	**Arteriolar RBF (nl/s)**	**0.49 ± 0.94** **^a^**	0.38 ± 1.19	0.752
	**Venular RBF (nl/s)**	**0.48 ± 0.93** **^a^**	0.35 ± 1.25	0.717
	**RTP (nl/s/mm^3^)**	0.37 ± 1.08	0.30 ± 1.03	0.848

NEDA, no evidence of disease activity; EDSS, Expanded Disability State Scale; LCLA, low contrast letter acuity; PERG, pattern electroretinogram; mRNFL, macular retinal nerve fiber layer; pRNFL, peripapillary retinal nerve fiber layer; PS-OCT, polarization-sensitive OCT; PR/UD, phase retardation per unit depth; OCTA, optical coherence tomography angiography; VVDr, volumetric vessel density of retinal vascular network; VVDs, volumetric vessel density of superficial vascular plexus; VVDd, volumetric vessel density of deep vascular plexus; UHR-OCT, ultra-high-resolution optical coherence tomography; TRT, total retinal thickness; RNFL, retinal nerve fiber layer; GCIPL, ganglion cell, and inner plexiform layer; RFI, retinal function imager; RBF, retinal blood flow; RTP, retinal tissue perfusion.

Δ, Differences between baseline and 1-year follow-up.

a With bold values, *P* < 0.05 baseline versus 1-year follow-up. Bold *P* value represents < 0.05.

b Only one eye of each patient was imaged.

In the non-NEDA subgroup, arteriolar RBF, venular RBF, and RTP were significantly increased at 1-year follow-up (*P* < 0.05). In the NEDA subgroup, significantly decreased superior PR/UD was observed at the follow-up visit (*P* < 0.05).

## Discussion

To the best of our knowledge, this is the first longitudinal prospective study that monitored a series of retinal microstructural, microvascular, and neuronal functional and axonal microstructural variables in patients with RRMS. No significant differences in EDSS and LCLA at the follow-up visit compared to that at baseline. The intriguing findings of this study were the improvement of retinal microcirculation (increased RBF and RTP) and the worsening of axonal microstructural integrity (decreased RNFL birefringence) at the follow-up visit in this cohort of patients with stable EDSS, LCLA, and thickness of RNFL.

In MS, the thinning of the RNFL and GCIPL is not only regarded as an early phenomenon,^34^^,^^35^ but also has been used as a biomarker for monitoring disease progression.^36^^,^^37^ However, the yearly thinning rate of the RNFL is within 1 µm,^38^ which may explain why we did not see significant changes in pRNFL thickness in the present study. In contrast, the axonal microstructural integrity tested as the birefringence showed a significant decrease, especially in the subgroup of patients who had increased disease activities (clinical relapses and/or radiological disease activity) and associated DMT escalation. The worsening of axonal microstructural integrity but preserved pRNFL thickness indicated possible subtle deterioration of the axonal filaments, such as neurofilaments, axoplasmic membranes, and microtubules.^13^ It coincides with the previous reports that deterioration of axonal cytoskeleton, including neurofilament compactness and phosphorylation, could precede axonal loss.^39^ The neurodegeneration in patients with MS is noted to be an early onset and continuous progression, even if there are no clinical signs of relapses.^40^^,^^41^ Hence, the decreased axonal microstructural integrity but preserved RNFL thickness indicate that RNFL birefringence may be a sensitive biomarker of neurodegeneration.

Furthermore, those patients who were in NEDA status and did not change their DMT had significantly improved retinal microcirculation (increased RTP). On the contrary, those patients who required DMT escalation due to the suboptimal response to treatment did not have significant improvement in their retinal microcirculation. These results imply that the retinal microcirculation might correlate with disease activities.

The low contrast visual acuity has been suggested to reflect the neurologic impairment in MS.^21^ Hence, the analysis of subgroups based on the change of the LCLA may provide some critical information. Indeed, in patients with stable or improved 1.25% LCLA, decreased VVDr and VVDs and increased BFV, RBF, and RTP were evident, indicating the improvement of the retinal microvasculature and microcirculation associated with improved visual function. Previous cross-sectional studies demonstrated that the increased VVD might be due to inflammation in patients with RRMS and was found to associate with poor low contrast visual acuity and disability.^17^ A trend of increased VVD from healthy subjects to patients with MS and to patients with a history of ON has been reported.^17^ The increased VVD has been speculated to be due to diffuse chronic inflammation and relate angiogenesis.^42^^,^^43^ The increased inflammation has also been confirmed by histopathologic studies in the brain and retina in patients with MS.^9^^,^^44^ The present study showed the recovery of retinal microvasculature (i.e. decreased VVD) occurred in patients with improved LCLA, suggesting that improved microvasculature might contribute to the improvement of visual function. On the other hand, the retinal microcirculation (abnormally decreased RBF and RTP) was reported to be impaired in patients with RRMS.^16^^,^^32^ In the present longitudinal study, some recovery (i.e. increase of the RBF and RTP) of retinal microvascular function was observed in these patients with stable and improved visual function. Furthermore, the recovery of retinal blood flow co-existed with the decrease of the VVD in patients with improved LCLA may indicate that RTP and VVD could be potential markers for evaluation of disease progression and therapeutic efficacy.

Interestingly, temporal and superior PR/UD measurements were significantly decreased at follow-up in patients with improved 2.5% LCLA. This finding is consistent with previous reports that temporal pRNFL (pRNFL-T)^45^^,^^46^ and nasal GCIPL^31^ were significantly thinner when comparing MS patients without a history of ON to healthy controls. The thinning in these sectors was found to associate with visual function and disability. Furthermore, the nasal GCIPL focal thinning of MSNON could be used to predict the conversion to RRMS from the clinically isolated syndrome.^47^ Hence, the decreased temporal and superior PR/UD measurement in patients with improved 2.5% LCLA could be due to the insidious progression in these clinically stable patients, indicating temporal pRNFL axonal microstructural integrity may be a sensitive biomarker in monitoring the disease progression and therapeutic efficacy. However, this speculation will need to be validated in future studies with a large sample size and longer follow-up period.

ON is highly prevalent in patients with MS.^48^ More subretinal thinning,^22^ impaired microvasculature^17^ and microcirculation,^16^^,^^32^ and RGC function^12^ have been well documented in patients with MS and a history of ON. Shortened PERG latency in patients with RRMS and more profound shortening in the eyes with a history of ON were demonstrated.^12^ The increased PERG latency in the eyes with a history of ON compared to the eyes without a history of ON, found in the present study, may indicate some recovery of the RGC function.

There are some limitations of the present study. First, the follow-up period may be too short, which may not show the slow and subtle changes. In addition, the follow-up window was too broad, which may further yield variation of the changes. Further studies with longer follow-up time and narrower windows are needed. Second, we did not have patients with progressive MS to further explore these retinal parameter alterations in disease progression. Third, although the MRI brain was used to define the disease activity, the brain volume loss was not measured. In addition, simultaneously studying the changes in the eyes and brain will further validate whether these retinal alterations could be developed as biomarkers for monitoring disease progression and therapeutic efficacy.

In summary, this longitudinal study of patients with RRMS revealed that the decline of the axonal microstructural integrity in the setting of preserved pRNFL thickness and stable LCLA, which indicated that RNFL birefringence could be a sensitive marker of neurodegeneration. Furthermore, the improved retinal vascular function occurred in patients with improved LCLA, and those in NEDA status suggested that these measurements may be developed as imaging markers for monitoring disease progression and therapeutic efficacy.
